# Integration of network-based approaches for assessing variations in metabolic profiles of alkalized and non-alkalized commercial cocoa powders

**DOI:** 10.1016/j.fochx.2024.101651

**Published:** 2024-07-15

**Authors:** Marta Palma-Morales, Oscar Daniel Rangel-Huerta, Caridad Díaz, Estela Castilla-Ortega, Celia Rodríguez-Pérez

**Affiliations:** a Department of Nutrition and Food Science, Faculty of Pharmacy, University of Granada, Cartuja Campus, 18011 Granada, Spain; b Institute of Nutrition and Food Technology (INYTA) 'José Mataix', Biomedical Research Centre, University of Granada, Avda. del Conocimiento s/n, 18071 Granada, Spain; c Section of Chemistry and Toxinology, Norwegian Veterinary Institute, P.O. Box 64, N-1431 Ås, Norway; d Fundación MEDINA Centro de Excelencia en Investigación de Medicamentos Innovadores en Andalucía, Avda. del Conocimiento 34, 18016 Armilla, Spain; e Biomedical Research Institute of Malaga and Platform in Nanomedicine-IBIMA Platform BIONAND, Málaga, Spain; f Department of Psychobiology and Methodology of Behavioural Sciences, Faculty of Psychology, University of Malaga, Málaga, Spain; g Instituto de Investigación Biosanitaria ibs.GRANADA, 18012 Granada, Spain

**Keywords:** Cocoa, Alkalization, Metabolomics, Molecular network, Chemometrics

## Abstract

Cocoa can undergo an alkalization process to enhance its color and solubility. It reduces astringency and alters its composition, particularly in the phenolic compound content, which is related to cocoa health benefits. This study aimed to investigate the impact of alkalization on the composition of seven commercial cocoa powders. A liquid chromatography-based metabolomic approach was employed to assess the metabolic differences between alkalized and non-alkalized cocoa powders. Supervised orthogonal partial least squares discriminant analysis (OPLS-DA) was used to identify the most discriminating variables between groups. A feature-based molecular network (FBMN) was used to explore the chemical space. Three hundred forty-seven metabolites were obtained as the most discriminant, among which 60 were tentatively annotated. Phenolic compounds, lysophosphatidylethanolamines, amino acids, and their derivatives were significantly reduced in alkalized cocoas. In contrast, fatty acids and their derivatives significantly increased with alkalization. Despite the variability among commercial cocoas, chemometrics allowed the elucidation of alterations induced specifically by alkalization in their composition.

## Highlights

• Alkalization reduced the phenolic compound content of cocoa.
• The content of fatty acids and their derivatives increased with alkalization.
• Alkalization reduced lysophosphatidylethanolamines, amino acids, and their derivatives.
• Advanced analytical techniques revealed the metabolic complexity of the cocoa.

## 1 Introduction

Cocoa is a widely distributed and globally recognized product. Its color, flavor, functional compounds, and established health benefits collectively contribute to its high regard as a favored food among consumers and industries. Cocoa beans are extracted and fermented from the harvested cocoa pods of *Theobroma cacao* to obtain nibs. The nibs, which are unshelled and fermented cocoa seeds, represent the final product of the primary cocoa production process. They are ground to obtain a paste called “cocoa liquor”. Cocoa liquor is pressed and divided into a solid part, known as “cocoa cake” and an oily part, “cocoa butter”. The cake is processed into “cocoa powder”. Cocoa powder is recognized for its light color, low solubility, and acidic, astringent, and bitter taste. Alkalization is a process used to enhance cocoa powder solubility; moreover it has the capacity to modify color and flavor. Incorporating an alkalization process darkens the color, reduces adverse sensory attributes, and enhances cocoa solubility (Valverde García, Pérez Esteve, & Barat Baviera, 2020). A darker color was found to be favorable in a study conducted with cocoa beverages, in which alkalized samples received the highest liking scores in terms of color (Palma-Morales, Rune, Castilla-Ortega, Giacalone, & Rodríguez-Pérez, 2024). During alkalization, cocoa is infused with an alkaline solution (usually NaOH and K_2_CO_3_ in concentrations from 1 to 6%) and it is exposed to precise temperatures (ranging from 60 to 130 °C) and pressures (varying from 0.10 to 1.22 MPa) for periods lasting from 5 to 180 min (Valverde García et al., 2020). Color changes result from various chemical reactions enhanced by alkaline conditions, oxygen, temperature, and pressure. These include the formation of Maillard reaction products, oxidation and polymerization of polyphenols, their interactions with other molecules, and the enhanced activity of polyphenol oxidase under basic conditions. Additionally, some authors have reported that alkaline solutions break ester links and hydrolyze cell walls. These effects, combined with high temperatures and pressures, lead to increased solubility through the destabilization and destruction of various complexes and cell structures (Valverde García et al., 2020). This process has also previously described to affect cocoa's composition.

(Goya, Kongor, & de Pascual-Teresa, 2022). Cocoa contains three primary categories of polyphenols, including flavanols such as catechin, epicatechin, and gallocatechin; anthocyanins (leucoanthocyanins and cyanidins); and proanthocyanidins (dimers, trimers, and other polymers of flavan-3-ols). To a lesser extent, cocoa also contains flavones, such as apigenin, luteolin, and kaempferol, alongside phenolic acids, such as caffeic and chlorogenic acids. These compounds are associated with a range of advantages for the cardiovascular system, including lowering insulin resistance and anti-inflammatory properties, but also with positive impacts on gut microbiota, and the improvement of cognitive function (Palma-Morales, Melgar-Locatelli, Castilla-Ortega, & Rodríguez-Pérez, 2023; Razola-Díaz et al., 2023). However, according to their structures and chemical modifications, polyphenols have different sensory characteristics and are recognized as pigmenting, astringent, and bitter compounds, with the capacity to alter the cocoa taste (Valverde García et al., 2020). Cocoa also contains methylxanthines, such as theobromine, caffeine, and theophylline, which influence its taste by conferring astringency and bitterness, and has also been associated with beneficial physiological and psychological effects (Goya et al., 2022). However, the composition of cocoa varies considerably depending on its genotype, geographic location, farming techniques, environmental factors, and post-harvesting operations, among other factors (Kadow, 2020; Palma-Morales et al., 2023; Razola-Díaz et al., 2023; Valverde García et al., 2020). Processing, particularly alkalization, can reduce the presence of phenolic compounds and methylxanthines, potentially affecting the health-related attributes of the cocoa (Goya et al., 2022). For the comprehensive study of cocoa composition and to understand how processing, such as alkalization, affect it, metabolomics emerges as a potent tool for the search for biomarkers related to the quality, authenticity, safety, and traceability of food products (Selamat, Rozani, & Murugesu, 2021). Targeted metabolomics approaches have been used to identify specific compounds of interest in cacao, such as polyphenols, catechins, flavonoids, and methylxanthines. A suitable method for analyzing the chemical composition forms the basis for comprehensively investigating the composition of cocoa. Liquid chromatography-tandem mass spectrometry (LC-MS/MS) has emerged as the primary technique for scrutinizing the chemical makeup of plant-based foods owing to its remarkable precision and sensitivity (Tong et al., 2021). While numerous studies have employed LC-MS/MS to analyze the chemical components in chocolate (de Dias et al., 2023) and cocoa beans (Bacca-Villota, Acuña-García, Sierra-Guevara, Cano, & Hidalgo, 2023), only a few articles have documented the comprehensive or the discovery of new compounds resulting from the alkalization process (Greño, Herrero, Cifuentes, Marina, & Castro-Puyana, 2022). Cocoa powders typically consist of a mixture of numerous compounds, resulting in complex mass spectrometry data after LC-MS/MS analysis, which difficult the interpretation. The use of molecular networking (MN) can aid in data interpretation. MN is a method for visualizing tandem MS/MS data that calculates the similarity degree of the MS/MS spectra and organizes them into a network, connecting compounds with similar structural elements through edges (Tong et al., 2021). This technique is valuable for analyzing complex chemical compositions, identifying quality, traceability, and safety biomarkers, and detecting contaminants and adulterants in foods (Li et al., 2023). It has become essential for examining the structures of compounds found in plant-based foods, enabling the identification of multiple unannotated compounds within a single group (Tong et al., 2021).

Thus, this study aimed to assess the metabolic differences between alkalized and non-alkalized (natural) cocoa powders using a non-targeted high-performance liquid chromatography-quadrupole time-of-flight mass spectrometry (HPLC-QTOF-MS)-based metabolomic approach. These differences in the metabolic profile may account for changes in the nutritional and sensory characteristics of cocoa as well as its bioactivity.

## 2 Materials and methods

### 2.1 Cocoa samples

Five non-alkalized cocoa powders from Venezuela (C1), Ivory Coast (C3), Peru (C4), Dominican Republic (C5), and West Africa (C6) and two alkalized cocoa powders from Ivory Coast (C2) and West Africa (C7) were purchased from the Spanish market. Commercial cocoa varieties that did not differ significantly in terms of nutritional composition were sought.

### 2.2 Sample preparation

Metabolites were extracted from cocoa powder using ultrasound technology as previously described by Razola-Díaz et al. (2023). First, the cocoa samples were defatted with hexane (10 ml hexane/ 1 g cocoa powder), vortexed for 1 min, sonicated in an ultrasound bath (Bandelin, Sonorex, RK52, Berlin, Germany) for 5 min, centrifuged (OHAUS, FC5718R, Germany) at 9960 rcf for 5 min, and evaporated under nitrogen. This procedure was repeated twice. Extraction was performed by adding 5 ml of a mixture of acetone/water/acetic acid (70/29.5/0.5%), vortexed for 2 min, sonicated in an ultrasound bath at a frequency of 35 kHz for 5 min, and centrifuged at 9960 rcf for 5 min. The extraction procedure was repeated twice, and the supernatants were collected, filtered with regenerated cellulose filters 0.2 μm (Millipore, Bedford, MA, USA) and stored at −18 °C until the analyses.

For the metabolomic sequence, each cocoa sample was extracted twice, and equal aliquots of each cocoa extract were mixed to prepare quality control (QC) samples to track the analytical performance.

### 2.3 Analysis

The analyses were performed using HPLC-QTOF-MS. Analytical separation was conducted by liquid chromatography (LC) using an Agilent series 1290 instrument (Agilent Technologies, Santa Clara, CA, USA) operating in reverse phase mode (RP). This process utilized an Atlantis T3 C18 column (2.1 mm × 150 mm, 3 μm particle size) from Waters (Waters Corporation, Milford, MA, USA). Mobile phase A contained a mixture of water/acetonitrile (90/10) and 0.1% formic acid, whereas mobile phase B contained acetonitrile/water (90/10) and 0.1% formic acid. The chromatographic run lasted 20 min. The gradient elution was as follows: 0.0–0.5 min with 1% eluent B; 0.5–11.0 min with 99% eluent B; 11.0–15.5 min with 99% eluent B; 15.5–15.6 min with 1% eluent B; and 15.6–20.0 min with 1% eluent B. Mass detection employed a Triple TOF 5600 QTOF-MS (SCIEX, Concord, ON, Canada). The mass spectrometer was operated using electrospray ionization in positive and negative modes and an information-dependent acquisition method, fragmenting the eight most intense signals. The exact mass calibration was performed automatically after every six injections. The two replicates of each sample were injected.

### 2.4 Data processing and multivariate analysis

Peak View software (version 1.1.2; AB SCIEX) was used to assess variations in retention time and mass-to-charge (*m/z*) ratios the three random peaks which presented a good shape at different time points and *m/z* values. This assessment was pivotal in establishing alignment ranges. Marker View software (version 1.2.1; SCIEX) facilitated peak detection, alignment, and data filtering. The collection parameters were configured with a 0.12 min retention time window, a noise threshold of 100 counts per second (cps), and a mass tolerance of 14.0 ppm (ppm). Moreover, only monoisotopic peaks were considered, thereby reducing mass duplication and enhancing the selection of authentic molecular features. The removal of contaminants and signals originating from solvents was achieved using blank samples. Raw data was converted into the open format *.mzML to make possible the analysis in third-party software.

Multivariate statistical analysis was performed using MetaboAnalyst 5.0 (https://www.metaboanalyst.ca). The data were centered and divided by the square root of the standard deviation as a Pareto scaling factor. Unsupervised principal component analysis (PCA) was performed to assess clustering among the different cocoa samples. Discriminating variables were determined using supervised orthogonal partial least squares discriminant analysis (OPLS-DA). The models were evaluated using the goodness-of-fit parameters, R^2^Y and Q^2^. Variable influence on the projection (VIP) values above 1.5 or below −1.5 were selected as significant and therefore the most important metabolites differentiating the groups of cocoa samples.

### 2.5 Metabolite identification and molecular networking

First, the differential features between alkalized and non-alkalized cocoas were annotated using SIRIUS 5 (https://bio..informatik.uni-jena.de/sirius/). Exact mass and isotopic patterns were utilized to generate the molecular formulas for the detected metabolites. Chemical classes, were then obtained using either the CANOPUS module (Dührkop et al., 2021) in SIRIUS 5 or using MS2query (v1.4.0) (de Jonge et al., 2023) to access to the NPClassifier classification tool (Kim et al., 2021), when there was no available class assigned, the ClassyFire most specific class was used. Only matches with a score above 0.66 were considered.

A feature-based molecular network (FBMN) was generated using the Global Natural Products Society Molecular Networking (GNPS2) (Wang et al., 2016). During this phase, raw data were processed using MS-DIAL 5 (http://prime.psc.riken.jp/compms/msdial/main.html), and a script was used to convert the file to the GNPS2 format (https://github.com/lfnothias/FBMN_MSDIAL5/blob/main/msdial5_formatter.py). The parameters for the FBMN in negative and positive ionization modes are available in the Supplementary Material (Table S1). Chromatograms are available in the Supplementary Material.

Both sets of data were cross-referenced to enrich the molecular network. Related compounds were linked based on the similarities of their MS/MS spectra, and the results were presented as molecular networks, aiding in the annotation process.

## 3 Results and discussion

### 3.1 Cacao powder chemical profile

**Fig. 1:**
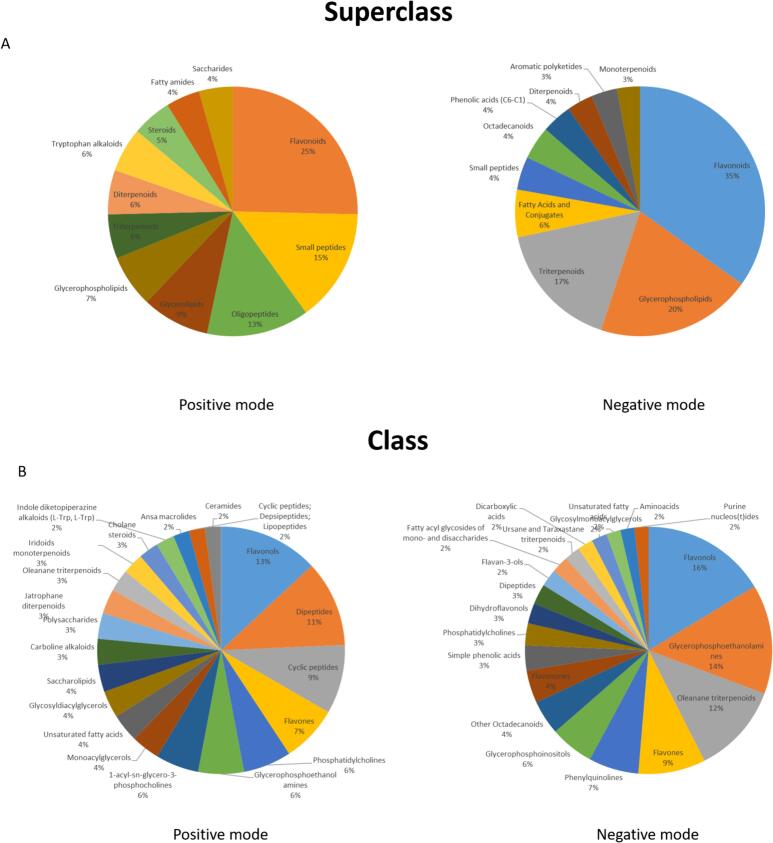
Summary of the A) Top10 chemical superclasses according to NPClassifier in the pool of the cocoa powder samples B) Top20 chemical superclasses according to the NPClassifier both in positive and negative ionization modes.

Prior statistical analysis, the cacao powder samples chemical space was explored using an automated approach based on MS/MS data from the pooled QCs using MS2query. Fig. 1A presents the top 10 chemical superclasses for positive and negative ionization modes found in the samples (see detailed list in Supplementary Tables S2 and S3). Several chemical superclasses appeared in both modes and their percentage was similar, whereas other classes were unique to a ionization mode as expected. Among those classes were flavonoids, small peptides, oligopeptides, glycerolipids, triterpenoids, diterpenoids, monoterpenoids, tryptophan alkaloids, steroids, fatty amides, saccharides, fatty acids and conjugates, octadecanoids and aromatic polyketides. On the other hand, Fig. 1B presents a deeper insight into the composition as it shows the top 20 NPC classes (or the most specific class according to the ClassyFire if NPC was not assigned) for each ionization mode on the cacao powder samples.

Overall, the exploration of the chemical space revealed that our analytical method was able to capture the high complexity of the cacao powder. Among the most relevant superclasses, tryptophan alkaloids, phenolic acids and flavonoids have been associated with many of the benefitial health related claims of cacao (Tan, Lim, Yeo, Lee, & Lai, 2021). Whereas peptides have been used as organoleptic quality indicators and biomarkers of the fermentation process but recent claims regarding their potential bioactive properties have arise and need further exploration (Domínguez-Pérez, Beltrán-Barrientos, González-Córdova, Hernández-Mendoza, & Vallejo-Cordoba, 2020). Furthermore, the presence of diverse fatty acids, glycerophospholipids and terpenoids could correspond to additives included in the cacao powder, as they have been suggested as adulteration biomarkers (Greño, Plaza, Luisa Marina, & Castro Puyana, 2023). When narrowing the view into the classes, besides the most known secondary metabolites in cacao, namely flavonols and flavones and flavan-3-ols, we observed the presence of many other secondary metabolites that might act have relevant roles both as bioactive compounds and quality biomarkers.

### 3.2 Multivariate statistical analysis

**Fig. 2:**
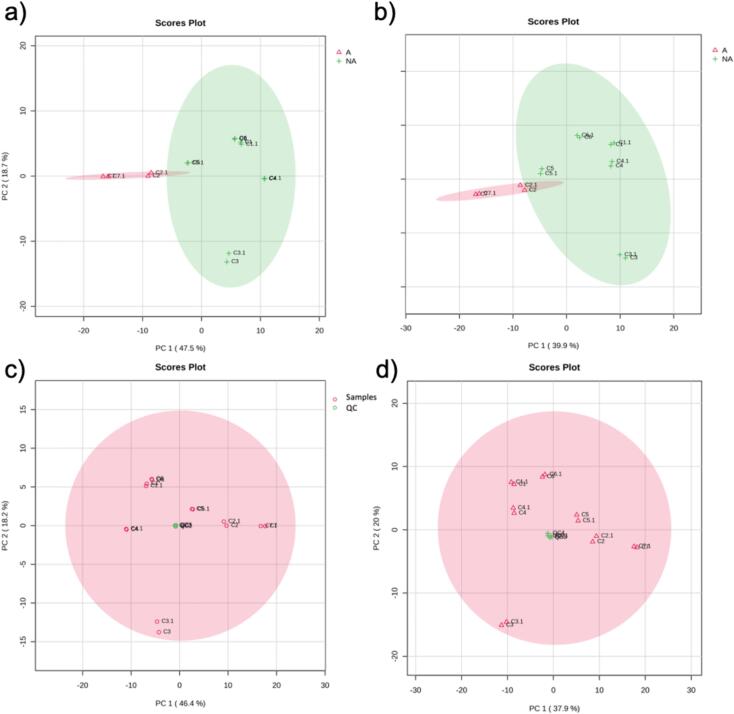
Principal Component Analysis (PCA) score plot obtained in the negative (a) and positive (b) ionization modes, and incluing QC samples in the negative (c) and positive (d) ionization modes. A: Alkalized; NA: non-alkalized.

After data pre-processing, filtration and quality control assessment, two datasets containing 872 features in the negative mode and 1335 in positive mode were used for further statistical analysis. PCA was selected to conduct the multivariate statistical analysis, as it condenses vast amounts of data into a simple model, facilitating the identification of clusters or sample groupings. PCA score plots for the positive and negative ionization modes are shown in Fig. 2**.** Alkalized and non-alkalized samples were clustered, and clear differentiation between them was achieved for both ionization modes. The first component of the PCA models explained 47.5% and 39.9% of the variance in the negative and positive ionization modes, respectively, according to the alkalization process. The second component of the PCA models explained 18.7% and 21.2% of the variance in the negative and positive ionization modes, respectively, probably according to the origin of the cocoas. QC samples were also clustered in the PCA, reflecting the good quality of the analysis (Fig. 2C, D).

### 3.3 Selection of discriminant variables and annotation

**Fig. 3:**
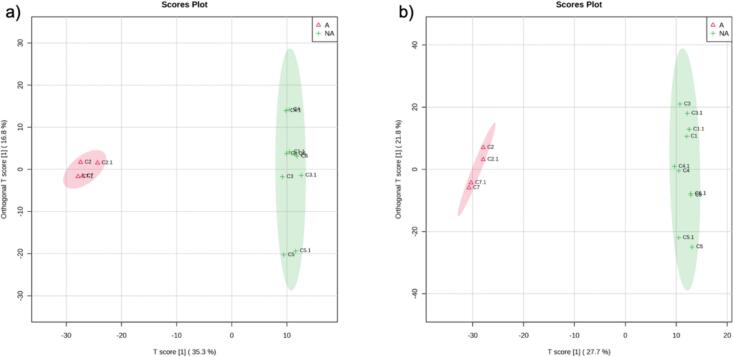
Orthogonal partial least squares discriminant analysis (OPLS-DA) obtained in the negative (a) and positive (b) ionization modes of alkalized vs. non-alkalized cocoa powders. A: Alkalized; NA: non-alkalized.

**Fig. 4:**
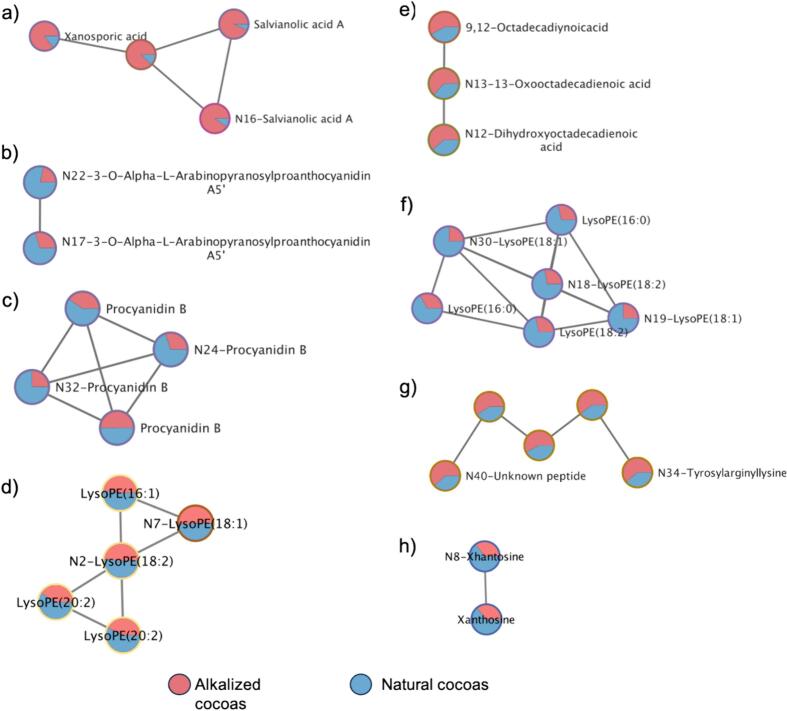
Feature-based molecular networks of some discriminating compounds, based on the OPLS-DA model, between alkalized and non-alkalized (natural) cocoa powders. Pie charts represent the relative abundance of each compound in natural and alkalized cocoa powders. a) N16; b) N16, N17; c) N24, N32; d) N2, N7; e) N12, N13; f) N18, N19, N30; g) N34, N40; h) N8. LysoPE: Lysophosphatidylethanolamine.

Once unsupervised analysis was performed, OPLS-DA was used to identify the most discriminating variables between the natural and alkalized cocoa groups (Fig. 3). The reliability was determined using the permutation metrics of the OPLS-DA model. The R^2^Y and Q^2^ values were 0.995 and 0.967 for the negative mode and 0.996 and 0.943 for the positive mode, respectively, indicating good prediction performance. The differential features between groups were screened according to a VIP > 1.5 or < −1.5. A total of 137 metabolites in the negative ionization mode and 210 in the positive ionization mode were obtained as the most discriminant, among which 23 were tentatively identified in the negative ionization mode (Table 1 and Table S4) and 37 in the positive ionization mode (Table 2 and Table S5). Table 1, Table 2 show the differential features present in the predictive component and thus attributed to the alkalization process (11 and 37 compounds, respectively), while Tables S4 and S5, available in the supplementary material, display the differential metabolites relevant to the orthogonal component attributed to the origin. In Table 1, Table 2, the features are sorted by VIP value and numbered consecutively. A summary of the experimental *m/z*, retention times, VIP values, molecular formula, mass error, main MS/MS fragments (sorted from highest to lowest intensity), and trend of the tentatively identified metabolites during the alkalization process is shown. Fig. 4 shows the tentatively identified compounds that were part of molecular subnetworks. Pie charts represent the relative abundance of each compound in natural and alkalized cocoa powders.

**Table 1:** Tentative identification of significant variables in negative ionization mode related to the alkalization process.

N	*m/z*	RT	V1	V2	Molecular formula	Adduct	ppm	Main fragments	Tentative identification	Alkalization trend
1	151.041	4.62	1.6	0.4	C8H8O3	[M-H]-	−4.7515	108.0220, 109.0291, 136.0173	3,4-Dihydroxyacetophenone	↑
2	476.2789	10.54	1.6	0.5	C23H44NO7P	[M-H]-	0.1828	–	Lyso PE (18:2)	↓
3	337.1521	4.02	1.6	0.4	C14H26O9	[M-H]-	−0.7592	59.0140, 89.0241, 119.0346	Butanoate derivative	↑
4	544.2661	10.56	1.6	0.4	C25H42NO7P	[M + CH2O2-H]-	−1.7311	476.2781, 279.2328	Lyso PE (20:5)	↓
5	271.0607	7.66	1.5	0.5	C15H12O5	[M-H]-	−3.9734	151.0033; 119.0503	Naringenin	↓
6	327.2163	8.11	1.5	0.3	C18H32O5	[M-H]-	2.4825	185.1168, 197.1199, 209.1181, 221.1201, 309.1979, 291.1961	Trihydroxyoctadecadienoic acid	↑
7	478.2964	11.59	1.5	0.2	C23H46NO7P	[M-H]-	7.6668	281.2498, 196.0374, 214.0487	Lyso PE (18:1)	↓
8	313.0943	3.72	1.5	0.7	C10H14N6O6	[M-H]-	6.4335	151.0410, 152.0417	Xanthosine	↑
9	415.1044	4.84	1.5	0.8	C21H20O9	[M-H]-	−2.0616	289.0703, 301.0720, 245.0818	3,5,7-Triacetylcatechin	↓
10	461.1687	4.67	1.5	0.7	C20H30O12	[M-H]-	7.0691	415.1608, 149.0457, 89.0238	Phenylethanoid	↑
11	144.0663	3.55	1.5	0.8	C6H11NO3	[M-H]-	−1.6434	100.2113, 68.0502, 98.0603	Butyrylglycine	↑

Lyso PE: Lysophosphatidylethanolamine; ↑: increase; ↓: decrease

**Table 2:** Tentative identification of significant variables in positive ionization mode related to the alkalization process.

N	*m/z*	RT	V1	V2	Molecular formula	Adduct	ppm	Main fragments	Tentative identification	Alkalization trend
12	313.2347	8.58	1.8	0.5	C18H32O4	[M + H]+	1.4495	–	Dihydroxyoctadecadienoic acid	↑
13	295.2274	8.6	1.7	0.6	C18H30O3	[M + H]+	1.9942	277.2141, 121.1001, 67.0546	13-Oxooctadecadienoic acid	↑
14	317.1016	4.44	1.7	0.4	C17H16O6	[M + H]+	−0.2986	–	Persicogenin	↓
15	368.3524	9.96	1.7	0.7	C23H45NO2	[M + H]+	0.9607	309.279	N-(2-hydroxyethyl)henicos-6-enamide	↑
16	495.1285	5.99	1.7	0.7	C26H22O10	[M + H]+	−5.8033	435.1058, 123.0432, 283.0585	Salvianolic acid A	↑
17	709.1768	4.65	1.6	0.3	C35H32O16	[M + H]+	−5.1347	557.1287, 425.0842, 299.0545, 577.1331	3-O-Alpha-L-Arabinopyranosylproanthocyanidin A5’	↓
18	478.2907	10.48	1.6	0.7	C23H44NO7P	[M + H]+	−1.7893	337.2734, 460.2816, 306.2792	Lyso PE (18:2)	↓
19	480.3084	11.55	1.6	0.5	C23H46NO7P	[M + H]+	−1.3864	339.2896, 462.2956	Lyso PE (18:1)	↓
20	287.0548	6.82	1.6	0.7	C15H10O6	[M + H]+	0.1237	153.0163, 241.0473	Luteolin	↓
21	689.2069	1.33	1.6	0.7	C33H36O16	[M + H]+	2.4354	527.1570, 509.1444, 407.1141	Tricin 4’-O-(Threo-Beta-Guaiacylglyceryl) Ether 7-O-Beta-D-Glucopyranoside	↓
22	709.1763	5.15	1.6	0.7	C35H32016	[M + H]+	0.8018	557.1259, 425.0876, 577.1335, 539.1149	3-O-Alpha-L-Arabinopyranosylproanthocyanidin A5’	↓
23	399.2335	1.83	1.6	0.5	C17H30N6O5	[M + H]+	4.1216	158.0927, 197.1283, 175.1165, 116.0688	Pyroglutamylisoleucylarginine	↑
24	579.1494	4.13	1.6	0.6	C30H26O12	[M + H]+	0.0298	409.0916, 127.0380, 427.1012, 291.0867	Procyanidin B2	↓
25	518.2408	1.45	1.6	0.8	C21H35N5O10	[M + H]+	6.2349	–	Val-Asp-Val-Ala-Asp	↓
26	180.101	1.44	1.6	0.6	C10H13NO2	[M + H]+	6.6345	117.0686, 145.0639, 115.0525, 163.0738	Salsolinol (biogenic amine)	↓
27	373.2808	1.87	1.6	0.6	C18H36N4O4	[M + H]+	0.8517	129.1017, 199,0718, 84.0793, 242.1864	Unknown tripeptide	↓
28	532.3484	10.91	1.6	0.8	C23H45N7O7	[M + H]+	−5.2471	261.2217, 353.2687, 243.2081	H-DL-Glu-DL-Lys-DL-Lys-DL-Lys-OH	↑
29	259.1272	1.5	1.6	0.7	C11H18N2O5	[M + H]+	3.4032	213.1249, 84.0794, 195.1127	Pyrosaccharopin	↓
30	480.3053	11.26	1.5	0.7	C23H46NO7P	[M + H]+	7.2538	339.2898	Lyso PE (18:1)	↓
31	175.1184	1.14	1.5	0.8	C6H14N4O2	[M + H]+	−1.2114	70.0642, 116.0692, 60.0550	Arginine	↓
32	579.1491	4.97	1.5	0.9	C30H26O12	[M + H]+	5.5033	409.0886, 127.0381, 427.1010, 247.0602	Procyanidin B2	↓
33	277.1418	1.41	1.5	0.9	C11H20N2O6	[M + H]+	1.5053	130.0852, 213.0859, 84.0808, 195.0775	Saccharopin (catabolite lys)	↓
34	466.2685	8.9	1.5	0.4	C21H35N7O5	[M + H]+	−8.6938	273.1986, 301.1910, 160.1110	Tyrosylarginyllysine	↑
35	282.1187	1.71	1.5	0.9	C11H15N5O4	[M + H]+	−3.2271	150.0762, 190.0690	Methyladenosine	↑
36	152.0557	1.6	1.5	0.7	C5H5N5O	[M + H]+	−6.2887	135.0288, 110.0337, 55.0282	Guanine	↑
37	360.3109	11.83	1.5	0.8	C20H41NO4	[M + H]+	−6.6478	342.3007, 282.2775, 264.2684, 265.2536	C2 Phytoceramide	↑
38	393.1975	1.54	1.5	0.9	C15H28N4O8	[M + H]+	−3.8665	86.0944, 270.1403, 288.1510, 376.1954	H-DL-Ser-DL-xiIle-DL-Ser-DL-Ser-OH	↓
39	295.2268	10.28	1.5	0.8	C18H30O3	[M + H]+	1.1473	277.2163, 67.0545, 91.0543	9-oxooctadeca-10,12-dienoic acid	↑
40	384.2833	6.77	1.5	0.5	C20H37N3O4	[M + H]+	1.8915	225.1960, 239.2101, 267.2069, 253.1889	Unknown peptide	↑
41	357.2089	1.47	1.5	0.8	C16H28N4O5	[M + H]+	0.8778	183.1117, 130.0862, 147.1125	Pyroglutamylvalyllysine	↓
42	241.086	7.36	1.5	0.8	C15H12O3	[M + H]+	−6.0174	171.0798, 145.0636, 91.0533	2,4-dihydroxychalcone	↓

Lyso PE: Lysophosphatidylethanolamine; ↑: increase; ↓: decrease

Alkalization is a very useful process for improving the acceptability of cocoa. In fact, in a study conducted with cocoa beverages, those prepared with alkalized cocoa powder received higher scores for color, flavor, texture, and overall acceptability compared to those prepared with natural cocoa (Palma-Morales et al., 2024). However, this process can affect the composition of cocoa.

Changes were observed in various compounds, including phenolic compounds, fatty acids and derivatives, lysophosphatidylethanolamines, amino acids and their derivatives, and peptides. Specifically, phenolic compounds were reduced in alkalized cocoas, as did lysophosphatidylethanolamines, amino acids, and their derivatives. In contrast, fatty acids and their derivatives tended to increase after alkalization. The alterations in cocoa composition are described below according to the chemical class and the ionization mode.

Based on their structures, polyphenols are known to exhibit different sensory and functional characteristics, acting as pigments, astringent and bitter compounds. Alkalization reduces astringency and bitterness, while degrading polyphenols, which are mainly responsible for this mouth feeling. Glycosylation of flavanols and their interaction with products of the Maillard reaction that occur during alkalization can lead to a reduction in astringency (Valverde García et al., 2020). Several phenolic compounds were found to be reduced in alkalized samples. In negative mode, the feature with *m/z* 271.0607 (N5) was tentatively identified as naringenin and confirmed by its chalcone family class. It has been previously described in cocoa (García-Díez et al., 2022). Naringenin is known to possess several biological effects, such as antioxidant, antibacterial and anti-inflammatory activities (Joshi, Kulkarni, & Wairkar, 2018). Feature N9, tentatively identified as 3,5,7-Triacetylcatechin, was confirmed by its chemical class (flavan-3-ols) and it is a derivative of catechin, which is a characteristic compound of cocoa. It has been reported that cocoa flavanol metabolites derived from catechin and epicatechin enhance glucose-stimulated insulin (Goya et al., 2022). In the positive ionization mode, features with *m/z* 317.1016 (N14) and *m/z* 287.0548 (N20) were tentatively identified as persicogenin and luteolin, respectively, and were confirmed by their chemical class. Luteolin is a flavone that has been previously described in cocoa (Díaz-Muñoz et al., 2023; Greño et al., 2023) and it possesses various beneficial effects on human health, including anti-diabetic, anti-hypertensive, anti-asthmatic and anti-viral activities (Punia Bangar, Kajla, Chaudhary, Sharma, & Ozogul, 2023). Nevertheless, feature N1 tentatively identified as 3,4-dihydroxyacetophenone, was increased in alkalized cocoas and has been previously described in cocoa (Chusniasih & Tutik, 2021). This compound has several beneficial activities, such as anti-inflammatory, antioxidative and cardio protective properties, as well as promoting coronary artery dilation and improving blood circulation (Cao et al., 2022). Salvianolic acid A (N16), was tentatively identified and found to increase in alkalized cocoas. It is a neolignane and potential fungal metabolite. It appeared in the same MN as xanosporic acid (Fig. 4a), an intermediate in the bacterial degradation of fungal toxins (Mitchell et al., 2003). Fungal contamination is inevitable during storage and processing, mainly during and after the fermentation step (Valverde García et al., 2020) and this could explain its presence. Features N17 and N22 were tentatively identified as 3-O-α-L-arabinopyranosylproanthocyanidin A5’ in Sirius and appeared in the same MN (Fig. 4b), suggesting they are isomers. Two isomers of Procyanidin B (N24 and N32) were tentatively identified and found in the same MN (Fig. 4c). Procyanidins are the main flavonoids contained in cocoa and have beneficial effects on the brain (Nehlig, 2013). The feature with *m/z* 689.2069 (N21) was tentatively identified as a phenolic glycoside, and the feature with *m/z* 241.0860 (N42) was doubly confirmed as dihydrochalcone.

Regarding changes in lipids, fatty acids and derivatives increased with alkalization, whereas lysophosphatidylethanolamines decreased in alkalized cocoas. Fatty acids and their derivatives have previously been found to increase with alkalization (Li et al., 2012), possibly due to the interaction between the alkali agent and triglycerides, leading to the hydrolysis of these compounds during processing (Valverde García et al., 2020). N2 was tentatively identified as lysophosphatidylethanolamine (18:2) and was reduced by alkalization, consistent with Greño et al. (2022). Additionally, it appeared in the same MN as lysophosphatidylethanolamine (18:1) (N7) (Fig. 4d), which was also reduced in alkalized cocoas. In the same line, feature N4, tentatively identified as lysophosphatidylethanolamine (20:5), was also reduced in the alkalized cocoas. Lysophospholipids (LPLs) are biosynthetic precursors of bioactive phospholipids with biological activity as lipid mediators. However, despite the growing interest in LPLs, further research is needed to determine if the LPL concentrations in cocoa are sufficient to induce health effects (Furukawa et al., 2016). On the contrary, feature N6, tentatively identified as trihydroxyoctadecadienoic acid and confirmed by its chemical class (octadecanoids), was increased in the alkalized cocoa powders. A butanoate derivative (N3) was also confirmed and increased in the alkalized cocoas. In positive ionization mode, features N12 and N13 were doubly confirmed as fatty acids by SIRIUS and GNPS, and they also appeared in the same MN (Fig. 4e). Both showed increased levels in alkalized cocoa powders, as fatty acid N6 identified in the negative mode did. Another feature (N39), classified by SIRIUS as a fatty acid derivative, also exhibited increased levels in the alkalized cocoas. Octadecanoids play functional roles in multiple biological processes, such as epidermal barrier formation, inflammation and immune modulation, metabolic processes, and cell proliferation. However, there remains a lack of knowledge about the biological activity of octadecanoids as lipid mediators (Quaranta, Revol-Cavalier, & Wheelock, 2022). Feature N15 was tentatively identified as a N-acyl amine, a class of conjugated fatty acid, and was increased by alkalization. Similarly, fatty acid amides have been found to increase with alkalization and have been proposed as markers of the alkalization process (Sirbu, 2018). Features N18, N19 and N30 were doubly confirmed as lysophosphatidylethanolamines and appeared in the same MN (Fig. 4f), showing a reduction due to the alkalization process, consistent with the findings of Greño et al. (2022).

The degradation of proteins through deamination and oxidation reactions during alkalization induced by temperature, enzymes, and an alkaline medium has been previously reported (Rodríguez, Pérez, & Guzmán, 2009). Amino acids and their derivatives tended to decrease during the alkalization process, except for butyrylglycine (N11), an amino acid derivative, which was increased in alkalized cocoas. Some peptides (features N23, N25, N27, N28, N34, N38, N40, and N41) were identified in positive ionization mode, with N34 and N40 appearing in the same MN (Fig. 4g). While some of these peptides were found to increase with alkalization, others decreased. The variations observed in protein behavior during the alkalization process can be attributed to the different conditions employed in this process, i.e., alkali concentrations or temperature, among others (Valverde García et al., 2020). Regarding amino acids, arginine (N31) showed reduced levels in alkalized cocoas, which agree with the findings reported by Greño et al. (2022). Arginine plays important roles in the body, including the production of nitric oxide and the removal of waste products (Nyawose, Naidoo, Naumovski, & McKune, 2022). The degradation of amino acids by alkalization was also described by Li et al. (2012). Similarly, decreased levels of pyrosaccharopine (N29) and saccharopine (N33), precursors of lysine, were found in alkalized cocoas. Lysine serves as a precursor to carnitine, essential for transporting fatty acids into mitochondria for energy production. It supports collagen formation, enhances bone health by reducing urinary calcium loss, and promotes its absorption (Aggarwal & Bains, 2022).

Other compounds were also identified in these samples. Feature N8, tentatively identified as xanthosine in the negative ionization mode, was also discriminant between both groups. It is a precursor of theobromine and caffeine. Xanthosine was confirmed by the MN in which it was immersed (Fig. 4h), and it increased in alkalized cocoas. Previous studies have shown that the levels of theobromine and caffeine decrease in alkalized cocoa (Urbańska, Derewiaka, Lenart, & Kowalska, 2019); therefore, we hypothesized that its precursor is increased in alkalized cocoa, likely due to the lack of biosynthesis of theobromine and caffeine during alkalization. A phenylethanoid (N10) was also identified and found to be increased in alkalized cocoas. In positive ionization mode, salsolinol (N26), a biogenic amine, was also tentatively identified. It has been previously found in chocolate and increased due to microbial fermentation (Díaz-Muñoz et al., 2023) and have shown neuroactive properties and thus often viewed as a neurotoxin (Chen et al., 2018; Villageliú, Borts, & Lyte, 2018). In the present work, it was found to be decreased in alkalized cocoa, whereas Greño et al. (2022) found no difference in this compound due to alkalization. Sioriki et al., 2022 observed a slight increase, but it was not statistically significant (Sioriki et al., 2022). The feature tentatively annotated as methyladenosine (N35) exhibited increased levels in alkalized cocoas, which is consistent with the trend observed in adenosine by Greño et al. (2022). Features N36 and N37 were also doubly confirmed as guanine and C2 phytoceramide, respectively, and both were increased in the alkalized cocoas. Guanine was previously described in cocoa by Herrera-Rocha et al. (2021).

To our knowledge, 20 of the 42 tentatively annotated features were described for the first time in cocoa. These include 4 phenolic derivatives: salvianolic acid, persicogenin, 3,5,7-triacetylcatechin, and 2,4-dihydroxychalcone; 2 lysophosphatidylethanolamines: Lyso PE (20:5), Lyso PE (18:1); 3 fatty acids and a derivative: trihydroxyoctadecadienoic acid, dihydroxyoctadecadienoic acid, 13-oxooctadecadienoic acid and a butanoate derivative; 3 derivatives of amino acids: butyrylglycine, pyrosaccharopin, and saccharopin; 5 peptides: pyroglutamylisoleucylarginine, Val-Asp-Val-Ala-Asp, H-DL-Glu-DL-Lys-DL-Lys-DL-Lys-OH, H-DL-Ser-DL-xiIle-DL-Ser-DL-Ser-OH and pyroglutamylvalyllysine; xanthosine and phytoceramide C2. Additionally, to the best of our knowledge, this is the first report of a decrease in salsolinol and an increase in guanine with the alkalization process.

Nevertheless, a limitation of our study is that annotation led us to identifications up to Level 2 or 3. This fact implies that there is a level of uncertainity and although robust computational tools were used, future work should be focused in validating the identity of these compounds.

The significance of this study lies in the application of metabolomics in combination with chemometrics and computational metabolomics tools, allowing for a thorough investigation of the metabolite profile of foods. By employing this approach, we can gain a comprehensive understanding of how various treatments, such as alkalization, impact the quality and safety of foods, as well as assess the preservation, transformation, or reduction of bioactive compounds that may have a positive effect on consumer health. Moreover, exploring the impact of alkalization on the metabolic profile of cocoa would enable us to optimize production processes and enhance the quality of the final product, thereby improving its overall quality, safety, and nutritional value. Other studies have investigated the effect of alkalization on the composition of cocoa by controlling the parameters of this process (Greño et al., 2022; Rodríguez et al., 2009), however, in the present work, the focus was on elucidating the effects of alkalization on different commercial cocoa powders, for which the specific parameters used in the process are unknown. This strengthens the results obtained, making them more applicable to consumers, as they reflect the commercial reality that can be found in the market. Despite the variability in the treatments applied and other factors, such as origin, certain trends in the composition of cocoa associated with the alkalization process were observed. Further research is needed to elucidate how other factors, such as the origin of cocoa, can affect its composition.

## 4 Conclusion

In summary, this study investigated how alkalization affects the compositions of different commercial cocoa powders. It is worth highlighting the advantage of using a non-targeted method, as it allows for a broad coverage of metabolites, enabling the identification of several secondary metabolites, such as polyphenols, alkaloids terpenoids or even fungal-related metabolites, lipids and amino acids. To the best of our knowledge, 20 metabolites have were tentatively characterized for the first time in cocoa. Despite the variability between commercial cocoas, changes in various metabolites have been identified between natural and alkalized cocoas, elucidating alterations induced by the alkalization process. Specifically, phenolic compounds, which are essential contributors to cocoa health properties, showed significant reductions in alkalized cocoas, consistent with previous studies. Lipid derivatives, peptides, and amino acids also exhibit fluctuations, reflecting the interaction between the alkalization process and cocoa composition. Generally, fatty acids and derivatives tended to increase with alkalization, whereas lysophosphatidylethanolamines, amino acids, and derivatives tended to decrease. Lastly, we report for the first time the reduction of salsolinol, a potential neurotoxin, after alkalization. Furthermore, this study highlighted the utility of advanced analytical techniques, multivariate statistical analysis and FBMN, to analyze, identify and annotate food compounds, thus revealing the complex metabolic signature of cocoa. Additionally, these techniques could be useful for identifyng biomarkers related to the alkalization process, which could be employed in cocoa authentication. Although FBMN is an emerging tool in foodomics, it can provide valuable insights into the chemical composition of food.

## Funding

This study was funded by Grant PID2020-114374RB-I00 funded by MCIN/AEI/10.13039/501100011033 (to C.R.-P. and E.C.-O.) and Junta de Andalucía-Consejería de Universidad, Investigación e Innovación—Project P21_00777.

## CRediT authorship contribution statement

**Marta Palma-Morales:** Writing – review & editing, Writing – original draft, Visualization, Methodology, Investigation, Formal analysis, Data curation, Conceptualization. **Oscar Daniel Rangel-Huerta:** Writing – review & editing, Visualization, Validation, Supervision, Methodology, Formal analysis, Data curation. **Caridad Díaz:** Writing – review & editing, Resources, Methodology. **Estela Castilla-Ortega:** Writing – review & editing, Resources, Project administration, Funding acquisition. **Celia Rodríguez-Pérez:** Writing – review & editing, Visualization, Validation, Supervision, Resources, Project administration, Methodology, Funding acquisition, Formal analysis, Data curation, Conceptualization.

## Declaration of competing interest

The authors declare that they have no known competing financial interests or personal relationships that could have appeared to influence the work reported in this paper.

## Data Availability

Data will be made available on request.

## Appendix A Supplementary data

Supplementary material Image 1
