# Supplementary material for: Integration of network-based approaches for assessing variations in metabolic profiles of alkalized and non-alkalized commercial cocoa powders
Source: Food Chem X. 2024 Jul 15;23:101651. doi: 10.1016/j.fochx.2024.101651 (PMC11324845; doi:10.1016/j.fochx.2024.101651)
Supplement: Supplementary file 1 — Supplementary material [file mmc1.docx]

**Table S1.** Parameters used in GNPS2 for the negative and positive modes

| **Name** | **Value** |
| --- | --- |
| Feature finding tool | MSDIAL5 |
| Fragment tolerance | 0.1 |
| Library analog search | 1 |
| Library min cosine | 0.7 |
| Library min matched peaks | 6 |
| Library topk | 1 |
| Networking max shift | 1999 |
| Networking min cosine | 0.7 |
| Networking min matched peaks | 6 |
| Normalization | None |
| Pm tolerance | 0.01 |

**Chromatograms negative ionization mode**

Sample 1

Sample 2

Sample 3

Sample 4

Sample 5

Sample 6

Sample 7

**Chromatograms positive ionization mode**

Sample 1

Sample 2

Sample 3

Sample 4

Sample 5

Sample 6

Sample 7

Tables S2a-b and S3a-b, present the results from the survey on MS/MS samples corresponding to the pooled QCs using ms2query in positive and negative modes, respectively.

| **Table S2a. Count of the NPClassifier superclasses found in cacao powder in negative mode** | |
| --- | --- |
| **NPC superclass** | **Count** |
| Flavonoids | 187 |
| Glycerophospholipids | 108 |
| Triterpenoids | 89 |
| Fatty Acids and Conjugates | 33 |
| Small peptides | 23 |
| Octadecanoids | 23 |
| Phenolic acids (C6-C1) | 21 |
| Diterpenoids | 18 |
| Aromatic polyketides | 18 |
| Monoterpenoids | 16 |
|  |  |

| **Table S2b. Count of the NPClassifier classes found in cacao powder in negative mode** | |
| --- | --- |
| **NPC class** | **Count** |
| Flavonols | 80 |
| Glycerophosphoethanolamines | 70 |
| Oleanane triterpenoids | 58 |
| Flavones | 43 |
| Phenylquinolines | 32 |
| Glycerophosphoinositols | 28 |
| Other Octadecanoids | 22 |
| Flavanones | 21 |
| Simple phenolic acids | 16 |
| Phosphatidylcholines | 14 |
| Dihydroflavonols | 13 |
| Dipeptides | 13 |
| Flavan-3-ols | 11 |
| Fatty acyl glycosides of mono- and disaccharides | 11 |
| Ursane and Taraxastane triterpenoids | 10 |
| Dicarboxylic acids | 10 |
| Glycosylmonoacylglycerols | 10 |
| Unsaturated fatty acids | 9 |
| Aminoacids | 9 |
| Purine nucleos(t)ides | 9 |

| **Table S3a. Count of the NPClassifier superclasses found in cacao powder in positive mode** | |
| --- | --- |
| **NPC superclass** | **Count** |
| Flavonoids | 168 |
| Small peptides | 97 |
| Oligopeptides | 88 |
| Glycerolipids | 58 |
| Glycerophospholipids | 45 |
| Triterpenoids | 38 |
| Diterpenoids | 38 |
| Tryptophan alkaloids | 38 |
| Steroids | 34 |
| Fatty amides | 29 |
| Saccharides | 29 |
|  |  |

| **Table S3b. Count of the NPClassifier classes found in cacao powder in positive mode** | |
| --- | --- |
| **NPC class** | **Count** |
| Flavonols | 81 |
| Dipeptides | 70 |
| Cyclic peptides | 56 |
| Flavones | 46 |
| Phosphatidylcholines | 39 |
| Glycerophosphoethanolamines | 37 |
| 1-acyl-sn-glycero-3-phosphocholines | 35 |
| Monoacylglycerols | 23 |
| Unsaturated fatty acids | 23 |
| Glycosyldiacylglycerols | 22 |
| Saccharolipids | 22 |
| Carboline alkaloids | 21 |
| Polysaccharides | 21 |
| Jatrophane diterpenoids | 20 |
| Oleanane triterpenoids | 18 |
| Iridoids monoterpenoids | 17 |
| Cholane steroids | 16 |
| Indole diketopiperazine alkaloids (L-Trp, L-Trp) | 15 |
| Ansa macrolides | 13 |
| Cyclic peptides; Depsipeptides; Lipopeptides | 13 |
| Ceramides | 13 |
|  |  |

In Tables S4 and S5, features non related to alkalization process are presented. The features are sorted by VIP value and numbered consecutively, and a summary of experimental m/z, retention times, VIP scores, molecular formula, mass error, and main MS/MS fragments (sorted from highest to lowest intensity) is provided.

| \| **Table S4.** Tentative identification of significant variables in negative ionization mode not related to the alkalization process \| \| --- \| | | | | | | | | | |
| --- | --- | --- | --- | --- | --- | --- | --- | --- | --- | --- |
| **N** | **m/z** | **RT** | **V1** | **V2** | **Molecular formula** | **Adduct** | **ppm** | **Main fragments** | **Tentative identification** |
| 1 | 203.0833 | 3.45 | 0.6 | 1.5 | C11H12N2O2 | [M-H]- | 3.7858 | 116.0494, 142.0656, 74.0243, 159.0942 | L-Tryptophan |
| 2 | 540.3312 | 10.94 | 0.6 | 1.5 | C24H50NO7P | [M+CH2O2-H]- | 3.9375 | 480.3083, 255.2331 | 1-Palmitoylglycerophosphocholine= LysoPC(16:0/0:0) |
| 3 | 540.3311 | 11.27 | 0.5 | 1.5 | C24H50NO7P | [M+CH2O2-H]- | 5.6402 | 480.3122, 255.2337 | 1-Palmitoylglycerophosphocholine= LysoPC(16:0/0:0) |
| 4 | 566.3478 | 11.72 | 0.4 | 1.5 | C26H52NO7P | [M+CH2O2-H]- | 2.6442 | 506.3256, 281.2493, 224.0693 | LysoPC(18:1/0:0) |
| 5 | 341.108 | 1.39 | 0.2 | 1.5 | C12H22O11 | [M-H]- | 0.7474 | - | Alpha-trehalose |
| 6 | 387.1151 | 1.39 | 0.2 | 1.5 | C13H24O13 | [M-H]- | 4.9484 | 341.1092, 179.0568, 89.0243, 119.0350 | Unknown disaccharide |
| 7 | 566.3502 | 11.44 | 0.2 | 1.5 | C26H52NO7P | [M-H]- | 9.1066 | 506.3292, 281.2497, 242.0805 | LysoPC(18:1/0:0) |
| 8 | 554.3467 | 12.21 | 0.2 | 1.6 | C25H52NO7P | [M+CH2O2-H]- | 4.5775 | 494.3247, 269.2465 | LysoPC(17:0/0:0) |
| 9 | 235.1087 | 3.25 | 0.1 | 1.6 | C12H16N2O3 | [M-H]- | 6.0571 | 87.0563, 147.0455, 192.0671, 164.0722 | Alanylphenylalanine |
| 10 | 568.3635 | 13.41 | 0.1 | 1.6 | C26H54NO7P | [M+CH2O2-H]- | 5.4849 | 508.3446, 283.2652 | LysoPC (18:0/0:0) |
| 11 | 278.0676 | 4.33 | 0.1 | 1.7 | C13H13NO6 | [M-H]- | 3.8455 | 119.0502, 162.0562, 216.0661, 132.0294 | N-(E-4-Coumaroyl)-Aspartate |
| 12 | 369.0287 | 4.65 | 0.1 | 1.5 | C15H14O9S | [M-H]- | 2.5294 | 289.0734, 137.0236, 216.9822 | Catechin-5-sulfate |
| LysoPC: Glycerophosphocholines | | | | | | | | | |

In the negative ionization mode, L-tryptophan (N1), and some peptides (N9, N11) were identified. Alpha-trehalose (N5), and another disaccharide (N6), were also confirmed and in the same molecular network. Additionally, several glycerophosphocholines were identified (N2, N3, N4, N7, N8 and N10), appearing in the same molecular network as some lysophospatidylethanolamines, suggesting similar chemical classes of these compounds. Feature N12 was identified as catechin-5-sulfate, and contrary to other phenolic compound, its content was not related to the alkalization process. The differences in the content of these compounds among the different cocoa powders could be attributed to the to the various origins of the cocoas.

| **Table S5.** Tentative identification of significant variables in positive ionization mode not related to the alkalization process | | | | | | | | | |
| --- | --- | --- | --- | --- | --- | --- | --- | --- | --- |
| **N** | **m/z** | **RT** | **V1** | **V2** | **Molecular formula** | **Adduct** | **ppm** | **Main fragments** | **Tentative identification** |
| 13 | 357.2997 | 16.22 | 0.4 | 1.5 | C21H40O4 | [M+H]+ | -1.8086 | 265.2518, 247.2415, 121.1000 | Monoolein |
| 14 | 286.1535 | 5.77 | 0.3 | 1.5 | C16H19N3O2 | [M+H]+ | -2.0384 | 130.0652 | L-Valyl-L-Tryptophan Anhydride |
| 15 | 402.3357 | 16.01 | 0.3 | 1.5 | C26H42NO2 | [M+H]+ | -1.407 | 121.0633, 138.0901 | N-(4-hydroxyphenethyl)oleamide (Metaloproteasa) |
| 16 | 313.2735 | 15.68 | 0.2 | 1.5 | C19H36O3 | [M+H]+ | -8.4957 | 57.0692, 71.0831, 257.2451 | Glycidyl Palmitate |
| 17 | 239.2356 | 15.69 | 0.1 | 1.5 | C16H30O | [M+H]+ | - | 57.0692, 55.0530 | 1-Cyclobutyldodecan-1-one |
| 18 | 403.2349 | 4.59 | 0.0 | 1.5 | C21H30N4O4 | [M+H]+ | -3.8734 | 188.0686, 205.0943, 72.0794, 199.1472 | Val-Trp-Val |

In the positive ionization mode, other compounds were identified as monoolein (N13), previously described in cocoa butter by Liu et al. (https://doi.org/10.1016/j.foodchem.2005.12.010), glycidyl palmitate (N16), a glycidyl ester contaminant of vegetable fats also previously described in cocoa products (https://doi.org/[10.1002/fsn3.523](https://doi.org/10.1002%2Ffsn3.523); <https://doi.org/10.1016/j.lwt.2022.113692>), and a fatty acyl (N17). An amino acid derivative (N14), a tripeptide (N18), and a metalloprotease (N15) were also identified. The differences in the content of these compounds among the different cocoa powders were not attributed to the alkalization process but could be due to the various origins of the cocoas.
